# TRR: Target-relation regulated network for sequential recommendation

**DOI:** 10.1371/journal.pone.0269651

**Published:** 2022-06-24

**Authors:** Weiqiang Di, ZhiHao Wu, Youfang Lin

**Affiliations:** School of Computer and Information Technology, Beijing Jiaotong University, Beijing, China; Education University of Hong Kong, CHINA

## Abstract

Item co-occurrence is an important pattern in recommendation. Due to the difference in correlation, the matching degrees between the target and historical items vary. The higher the matching degree, the greater probability they co-occur. Recently, the recommendation performance has been greatly improved by leveraging item relations. As an important bond imposed by relations, these connected items should have a strong correlation in the calculation of certain measures. This kind of correlation can be the biased knowledge that benefits parameter training. Specifically, we focus on tuples containing the target item and latest relational items that have relations such as ***complement*** or ***substitute*** with the target item in user’s behavior sequence. Such close relations mean the matching degrees between relational items and historical items should be highly affected by that of the target item and historical items. For example, given a relational item having relation ***complement*** with the target item, if the target item has high matching degrees with some items in user’s behavior sequence, this complementary item should behave similarly for the co-occurrence of complementary items. Under guidance of the above thought, in this work, we propose a target-relation regulated mechanism which converts the biased knowledge of high correlation of matching degrees into a regulation. It integrates the target item and relational items in history as a whole to characterize the matching score between the target item and historical items. Experiments conducted on three real-world datasets demonstrate that our model can significantly outperform a set of state-of-the-art models.

## Introduction

**Fig 1:**
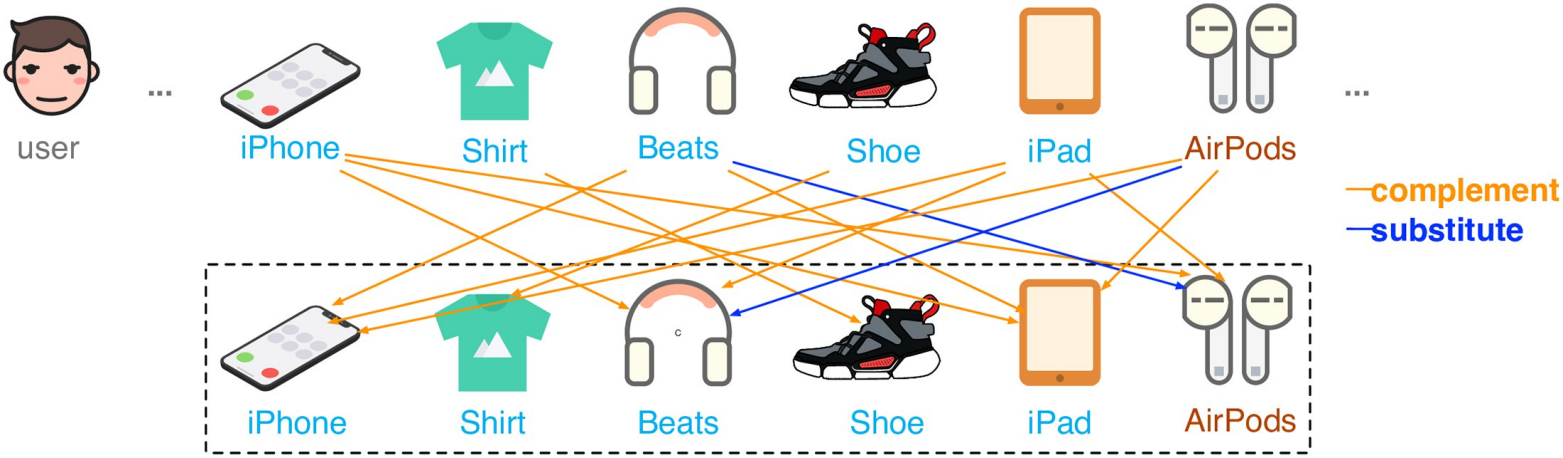
An example of user’s purchasing sequence with item relations.

Due to the overwhelming data that people are facing on the Internet, recommendation is becoming increasingly important. It can help to alleviate the problem of information overload in fields like e-commerce for retrieving information and discovering contents. Most existing recommendation models work on implicit feedback like purchasing records to learn personalized preference. Although effective, it is very challenging to further improve the performance due to the problem of data sparsity. Recently, some works have taken item relations into account and an example illustrating such relations is shown in Fig 1. Item relations like ***complement*** and ***substitute*** can help narrow the scope of recommendation, which greatly alleviate the data sparsity since you most likely will not buy an item if you have bought something similar in function recently. Based on such fine-grained information, models using such information have been studied to improve recommendation accuracy. The two prominent ones in them are CFKG [1] and Chorus [2]. CFKG is a pioneer work in mining item relations with knowledge graph. Chorus focuses on item relations and their temporal effects. Although the influence of item relations is considered, they use only the relation type information. It is somewhat indistinguishable for that the same relation can contain a large number of different items. Therefore, we now turn our attention to relational items of the target, which are historical items that have a relation with the target item. Compared to relation type, greater discrimination can be got with relational items since items can have different influences, strong or weak, even they may belong to the same relation. Besides, relational items are homogenous with target item since they all represent items, and this consistency helps in the measure of matching degree. However, this is not the case with relation types.

Relational items, which have a relation with the target item in user’s behavior sequence, can play a significant role in this scenario for their intimacy with the target and their potential remains to be further explored. Some characteristics between items that have relations are correlated. This feature can be viewed as a strong biased knowledge and converted to a regulator in terms of matching degree in the parameter learning. We are concerned about matching degrees between items to make decisions when doing recommendation, which is usually calculated by the dot product of their embeddings. For example, if the target item co-occurs often with some historical items, their matching degrees should be high. And the matching degrees between the target and irrelevant historical items should be low. As an item that has an important relation with the target, the matching degrees between this relational item and historical items should be strongly influenced by matching degrees between the target item and historical items. Depending on specific relation, their matching degrees may follow the same or opposite trends. For instance, given a relational item having ***complement*** relation with the target, the probability it co-occurs with the target item in user’s behavior sequence is much larger in average than other items that have no relation. If the target item has a high matching degree with a historical item A, where high matching degree also means large probability to co-occur, the relational item’s matching degree with item A should also be high because of the transitivity of co-occurrence.

Inspired by the above idea, in this paper, when evaluating the matching scores between the target and historical items, we replace the original target by integrating the target and relational item as a whole to characterize the matching score. This regulated matching score is then used to determine whether the target item is the one we are interested in the next step. These steps convert the aforementioned bias knowledge to the constraint in matching score to better employ such correlation.

In summary, the contributions of this work are as follows:

- We highlight the strong correlation between the target item and relational items can be viewed as a biased knowledge and converted to a regulator embedded in the parameter learning.
- We propose a target-relation regulated network which introduces a constraint by integrating the target and relational item as a whole to characterize the matching scores between the target and historical items.
- We evaluate our model on three real-world datasets and achieve significant improvements over the state-of-the-art baselines for recommendation.

## Related work

In this section, we briefly review the related works in two aspects, namely sequential recommendation and item relation modeling.

### Sequential recommendation

Recommendation systems have made considerable progress in recent years. The trajectory can be traced from the past Collaborative Filtering (CF) to the current Sequential Recommendation (SR) [3], which relies on the user’s behavior sequence to predict the next item that the user might be interested in. Explicit feedback such as ratings was modeled most in the early days. Models like MF [4] and SVD++ [5] has shown a very powerful representation ability. The research focus has transited from explicit feedback to implicit feedback later for their commonness and universality. The training content has also shifted from rating task to ranking task and finally evolved into the current top-k recommendation. Models like MF linearly aggregates the multiplications of latent embeddings, which is insufficient to capture complex user-item interactions. NCF [6] is thus proposed to learn non-linear function via a multi-layer neural network. Sequential patterns is the focus sequential recommendation wants to capture. Recurrent Neural Network (RNN) and its variants like Gated Recurrent Units (GRU) are incorporated into sequential modeling [7]. However, RNN has some shortcomings, such as difficulty in capturing long-term dependency, poor parallelism, and too strict order assumptions for interaction sequence. Subsequently, some Convolutional Neural Networks (CNN) have also been explored and obtained good results [8]. One of the problems of CNN-based models is that they have difficulties in capturing relations between items that are not nearby. Recently, there are works that employ advanced techniques, e.g., attention mechanism [9–12] and gating mechanism [13] for sequential recommendation to distinguish the importance of different items in sequence. SASRec [14], based on self-attention mechanism, demonstrated promising results in modeling mutual influence between historical interactions. HGN [13] exploits item co-occurrence as one of the model’s building block.

### Item relation modeling

Traditional recommendation techniques can perform well when sufficient interaction information is provided. However, we often encounter the problem of data sparsity in practice. In real-world, some relations with concrete semantics exist among items. To introduce more effective information and address the data sparsity, models incorporating item relations have recently got research attention [2, 15–17]. They mainly use Knowledge Graph (KG) to learn relation semantics between items and embed them to item embeddings. CFKG [1], which defines a variety of entities and relations, learns the representation over a structured heterogeneous knowledge graph for recommendation. Chorus [2] considers relation types between items and their corresponding temporal dynamics to better capture the evolutional effects of relations. There is a drawback here, which is that it needs handcrafted forms of temporal decay functions. KDA [18] makes better by introducing Fourier transform to model the varying temporal effects of different relational interactions.

## Methodology

### Problem formulation

We first formulate the task of sequential recommendation with item relations here. Let U and I denote the set of users and items respectively. All users’ interaction history $A=\{S^{1},S^{2},\ldots ,S^{\left|U\right|}\}$ are given. Each user has an interaction sequence of items happened in the chronological order $S^{u}=\left\{s_{1}^{u},s_{2}^{u},\ldots ,s_{t}^{u}\right\}$, where $s_{i}^{u}\in I,0\leq i\leq t$. The task is to choose k items from I that most likely to be of interest to the user based on the historical interactions at time step *t* + 1. Besides, in the task of knowledge graph embedding happened in the first part of our model, we denote R to be the set of item relations with size *h*, where relation r∈R could be ***complement*** or ***substitute***, etc.

### Model overview

**Fig 2:**
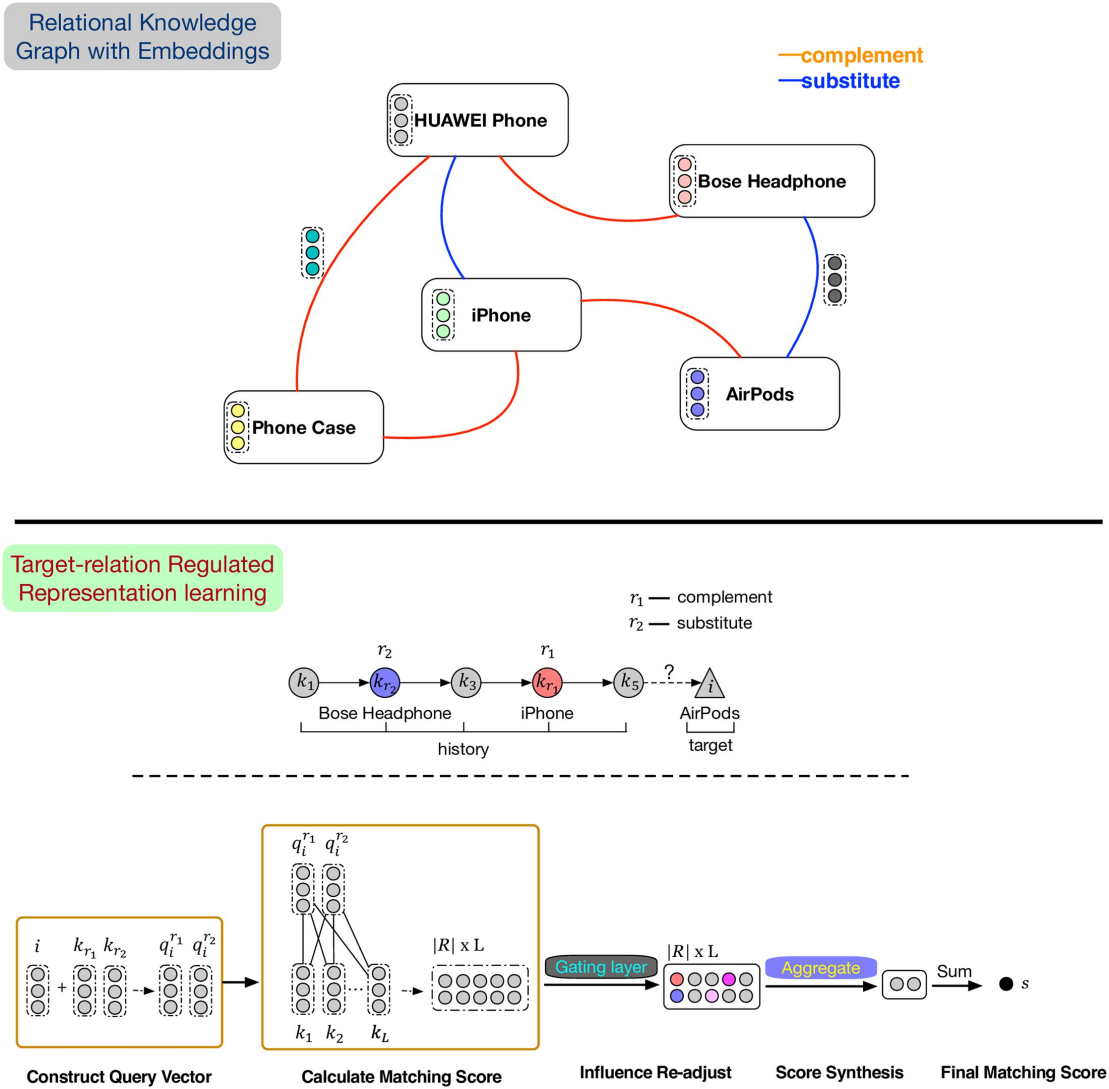
Illustration of the proposed model TRR. The first part pays attention to encoding the structural information constructed by the knowledge graph of item relations into their embeddings. The second part focuses on learning the target-relation regulated representation for the recommendation task based on the embeddings learned from the first part.

Fig 2 illustrates the overall architecture of TRR which consists of two parts. The first part is for item relation modeling, where we learn item representations from the knowledge graph of item relations and make a good initialization for the subsequent recommendation part. The second part is for recommendation task where the relational items are exploited to measure matching scores between the target and historical items.

### Relational knowledge graph embedding

Let’s first look at the task of item relation modeling from knowledge graph of item relations. There are often relations between items that we can employ. Take two commonly seen behaviors “*also_view*”, “*also_buy*” in shopping sites as an example. We denote “*also_view*” as relation ***substitute*** and “*also_buy*” as relation ***complement***, which are useful for accurate recommendation. For instance, if you have purchased an iPhone, it is very likely that you will show interest to AirPods for that a strong complementary relation exists between them. However, if you have purchased Powerbeats, you most likely don’t want to buy AirPods within a short period because they are functionally overlapping and substitutable. Items and relations between them can form a knowledge graph consisting lots of triples {*s*, *r*, *o*}, where *s* and *o* are items and r∈R is a relation. After getting the knowledge graph, the next step is how to train model parameters employing it. Considering the number of relations is small in this knowledge graph, we select TransE [19] from traditional knowledge graph embedding models as the training method. The score function for each triple used in TransE is:

$$\begin{array}{c} f\left(s,r,o\right)=\|s+r-{o\|}_{2}^{2} \end{array}$$ (1)

where **s**, **r** and **o** are embeddings of *s*, *r* and *o* respectively. The loss function for this task is:

$$\begin{array}{c} loss=-\text{log}\;\sigma \left(\gamma -d\left(s,o\right)\right)-\sum_{i=1}^{n}\frac{1}{k}\text{log}\;\sigma \left(d_{r}\left(s_{i}^{\prime },o_{i}^{\prime }\right)-\gamma \right) \end{array}$$ (2)

where *γ* is a fixed margin, *σ* is the sigmoid function, and $\left(s_{i}^{\prime },r,o_{i}^{\prime }\right)$ is the *i*-th sampled negative triplet.

### Training of knowledge graph

We have constructed a knowledge graph and corresponding loss function focusing on item relations in the previous step. Then we train the knowledge graph to learn item representations affected by item relations. After enough epochs of training, we turn to the second part of our model: the recommendation task. At that time, item embeddings have integrated the structural information from knowledge graph which cannot be learned in the subsequent recommendation task.

### Embedding layer

As shown in Fig 2, the input of TRR is a user’s behavior sequence, which contains a series of items in time order. We first convert the input $\left(s_{1}^{u},s_{2}^{u},\ldots ,s_{t}^{u}\right)$ into a fixed-length sequence *s*^*u*^ = $\left(s_{1}^{u},s_{2}^{u},\ldots ,s_{L}^{u}\right)$ to facilitate subsequent operation, where *L* denotes the maximum sequence length TRR will process. If the sequence length is greater than *L*, we truncate it and take the most recent *L* items, otherwise, we pad the sequence to the fixed length *L*.

We maintain an item embedding matrix $M^{I}\in R^{|I|\times d}$, where *d* is the embedding dimension. The item embedding matrix projects the high-dimensional one-hot representation of an item to a low-dimensional dense representation. Given an interaction sequence *s*^*u*^ = $\left(s_{1}^{u},s_{2}^{u},\ldots ,s_{L}^{u}\right)$, we can form the input embedding matrix:

$$\begin{array}{c} E=\left(k_{1},\ldots ,k_{L}\right)\in R^{L\times d} \end{array}$$ (3)

where $k_{r}\in R^{d}$ is the embedding of the *r*-th item.

### Target-relation regulated representation learning

Sequential dependency is ubiquitous in user-item interactions. To better determine users’ current interests, we should look into their historical interactions. However, we must be aware that in many cases, only a few items in the past will play an important role. How do we distinguish which items are important? Given a piece of interaction history for a user, some historical items have no relation with the target item and others have. Historical items that have important relations with the target item should undoubtedly deserve our attention. Because of the close relationship within the target and relational items, the matching degrees between the target item and historical items have a deep correlation with the matching degrees between the relational items and historical items. This can be viewed as biased knowledge and converted to a regulator in the calculation of matching scores.

For example, given two users’ purchasing history *U*_1_ = {Shoes, MacBook, Milk} and *U*_2_ = {Bose Headphone, Samsung Phone, HuaWei Watch}. Suppose we now predict whether they will be interested in buying an iPhone. By browsing their purchasing history, we can find the target iPhone is complementary with MacBook in *U*_1_, and a substitutable relationship with Samsung Phone in *U*_2_. Obviously, *U*_1_ is likely to be interested in iPhone while *U*_2_ most likely won’t because it already has a functionally similar one. The above scenario explains the importance of introducing relational items. Now we further elaborate on capturing the item co-occurrence by exploiting relational items. Given another user’s interaction history *U*_3_ = {MacBook, AirPods, Milk}. Compared with *U*_1_, *U*_3_’s willingness to buy an iPhone will be greater for that the union of two complementary items MacBook and AirPods leads to greater possibility on buying the target iPhone than just one complementary MacBook in *U*_1_.

Given the representation of the target item denoted as ***i***. In our mechanism, a strong correlation exists in the measure of matching degrees among the target item and items having relations with the target. To integrate such constraint, we first add the target item with the latest maximum *n* relational item in each type of relation from history as the query vector:

$$\begin{array}{c} q_{i}^{r}=i+\sum_{i=1}^{n}\lambda_{i}^{r}*j_{i}^{r} \end{array}$$ (4)

where r∈R is one of the item relations, $j_{i}^{r}$ denotes the embedding of historical item having relation *r* with the target item, $\lambda_{i}^{r}$ control the strength of each relational item and $q_{i}^{r}$ represents the query vector with respect to relation *r*. We list three ways here to aggregate the influence of multiple relational items. The first is to calculate the average, which sets coefficient $\lambda_{i}^{r}$ to be 1/*n*. In the remaining two, $\lambda_{i}^{r}$ is learned through a multilayer perceptron. Given the relational items $J=\left(j_{1}^{r},\ldots ,j_{n}^{r}\right)\in R^{n\times d}$. We can obtain the aggregated representation by the following three formula:

$$\begin{array}{c} \sum_{i=1}^{n}\lambda_{i}*j_{i}^{r}=\sum_{i=1}^{n}\frac{1}{n}*j_{i}^{r} \end{array}$$ (5)

$$\begin{array}{c} \sum_{i=1}^{n}\lambda_{i}*j_{i}^{r}=SUM(softmax\left(J^{⊤}W\right)*J^{⊤},2) \end{array}$$ (6)

$$\begin{array}{c} \sum_{i=1}^{n}\lambda_{i}*j_{i}^{r}=SUM(\sigma \left(J^{⊤}W\right)*J^{⊤},2) \end{array}$$ (7)

where $W\in R^{n\times n}$ is the trainable parameters. The superscript ⊤ denotes the matrix transpose. *SUM*(*A*, *i*) means we sum up the embeddings along the *i*-th dimension of *A* and *σ* is the sigmoid function. TRR adopts one special case, where *n* = 1 and λ_1_ = 1. This means only the latest relational item is used for each type of relation. The impact of different aggregation methods with λ_*i*_ will be explored in the ablation experiment. Then we use query vector $q_{i}^{r}$ to calculate the matching degrees with items in user’s behavior sequence. We use dot product to compute the matching degree:

$$\begin{array}{c} v_{m}^{r}=q_{i}^{r}\cdot k_{m} \end{array}$$ (8)

where *m* ∈ [1, *L*] represents the item position in user’s history sequence, vector ***k***_*m*_ is the item embedding in that position and $v_{m}^{r}$ is the matching degree between $q_{i}^{r}$ and historical item *k*_*m*_. The larger $v_{m}^{r}$ is, the higher probability of this item co-occurring with the target and latest relational item. To reduce noise introduced by irrelevant historical items, we further use a gating layer to adjust their relative matching degrees and then aggregate them with addition:

$$\begin{array}{c} \begin{array}{cc} & g^{r}=\sigma \left(W_{1}v^{r}\right)\\ & z^{r}=g^{r}\cdot v^{r} \end{array} \end{array}$$ (9)

where $W_{1}\in R^{L\times L}$, $v^{r}\in R^{L}$ with $v_{m}^{r}$ as one element in this vector, *σ* is the sigmoid function and *z*^*r*^ is the final adjusted matching degree between the target and historical items under a certain relation. Note that sigmoid is used here instead of softmax because there may be multiple items that are important. Using softmax will reduce the distinction between important items and non-important items. At last, we add up the influence from different relations to synthesize them:

$$\begin{array}{c} \begin{array}{cc} s & =\sum_{r\in R}z^{r} \end{array} \end{array}$$ (10)

### Prediction layer

We have introduced the core mechanism of TRR. Next we describe the prediction layer which combines matrix factorization and the above schema to produce the prediction score.

Since each user or item has a latent representation, the score function for ranking is as the following:

$$\begin{array}{c} {\hat{y}}_{ui}=u^{T}i+s \end{array}$$ (11)

where ***u***^*T*^***i*** gets the matching degree from the user-item perspective and the second *s* pays attention to the influence from the item-item perspective.

To train parameters in the recommendation part of TRR, we need to select an appropriate loss function to optimize. Since the user interactions are sequence of implicit feedback, pair-wise ranking loss is used to optimize the proposed model in training. It aims to rank the observed next item (positive) ahead of the accompanying negative sample and is formulated as:

$$\begin{array}{c} L_{rec}=-\sum_{u\in U}\sum_{i=1}^{N_{u}}(log\sigma \left({\hat{y}}_{ui}-{\hat{y}}_{uj}\right) \end{array}$$ (12)

where $j\notin S^{u}$ is a negative item sampled from the training set.

### On comparison with Chorus

Note that our proposed TRR is different from Chorus [2]. Chorus focuses on the temporal dynamics of item relations, where the representation of each item includes the basic item embedding and temporal evolution from different relations. However, our TRR doesn’t contain the time factor for that it has limited stacking effect on our mechanism. Another main difference is that, although Chorus considers the temporal influence of relations between items, it only uses the relation type information. However, it is somewhat vague since the same relation can contain a large number of different items, which leads to low discrimination. In contrast, TRR takes care of relational items of the target item. Greater discrimination can be got since different items have their own representations and contribute different influences, strong or weak, even they all belong to the same relation. Besides, compared to relation type, relational items are homogenous with the target item. This consistency helps in the measure of matching degrees. The third main difference is that Chorus mainly relies on taking both relation types and corresponding temporal dynamics into consideration to obtain performance improvement. Our TRR takes advantage of the strong correlation between the target and relational items as the biased knowledge and converted to a constraint in the calculation of matching scores to help learning parameters.

## Evaluation

In this section, we conduct experiments to verify the effectiveness of the proposed TRR. We first describe the datasets, evaluation metrics, baseline methods and experimental settings in detail. Then we report the experimental results and conduct a in-depth analysis.

### Datasets

To evaluate performance of the proposed TRR, we do experiments on three public amazon datasets. These datasets not only have the interaction records of users with timestamps, but also the metadata of items. Typical information in metadata contains relations between items like “*also_view*”, “*also_buy*” and the category information. We have selected 3 representative datasets from the amazon dataset library: *Grocery* *and* *Gourmet* *Food* (Grocery), *Cellphones* *and* *Accessories* (Cellphones), and *Home* *and* *Kitchen* (Home). The raw data and preprocessing code can be found in github (https://github.com/THUwangcy/ReChorus/tree/SIGIR20/data). Statistics information about the three datasets are summarized in Table 1.

**Table 1:** Statistics of the datasets.

Dataset	#Users	#Items	#entry	#triplet	relational ratio in test set
*Grocery*	14.7k	8.5k	145.8k	372.1k	27.8%
*Cellphones*	27.9k	10.3k	193.2k	247.5k	30.0%
*Home*	66.5k	27.2k	541.6k	924.6k	16.6%

### Evaluation protocols

To evaluate the top-N recommendation performance, we employ Hit Ratio (HR) and Normalized Discounted Cumulative Gain (NDCG) to measure the recommendation quality as done in many methods. HR@K indicates whether the test item successfully appear in the top-*k* recommended list and NDCG@K takes the ranking position of correctly recommended items into account.

### Comparison methods

To show the effectiveness of the proposed TRR, two groups of baselines are considered. The first group is the general sequential recommendation methods like SASRec [14] and HGN [13]. The other one is models employing item relations like Chorus [2] and KDA [18]. The compared state-of-the-art models are listed as the following:

- **BPR**. A classic CF method applying Bayesian Personalized Ranking to Matrix Factorization [4] for recommendation.
- **GMF**. A classic CF model using multiple non-linear layers of neural network [6].
- **Tensor**. A model splitting time into bins and factorizes a three-dimensional tensor for recommendation [20].
- **GRU4Rec**. A RNN-based model using gated recurrent unit for sequential recommendation [7].
- **NARM**. A RNN-based model combing GRU and attention for session-based recommendation [3].
- **Caser**. A CNN-based model which learns high-order dependency via vertical and horizontal convolutions for sequential recommendation [8].
- **SASRec**. A self-attention based model which can learn long-term dependency and identify relevant items for prediction [14].
- **HGN**. A attentive model which proposes a hierarchical gating architecture and the Item-item Product mechanism [13].
- **CFKG**. A collaborative filtering model learning over a structured knowledge graph [1].
- **SLRC**. A sequential model considering both item relations and their temporal dynamics [21].
- **Chorus**. A sequential model considering not only item relations but also their evolutional effects along time [2].
- **Locker**. A sequential model which improves self-attentive mechanism by enhancing short-term user dynamics modeling. [12].
- **KDA**. A sequential model considering relational effects and their temporal evolutions using Fourier transform [18].

### Implementation details

For comparison purpose, we follow some configurations used in Chorus [2]. The embedding size is set to 64. We use Xavier initializer with a mean of 0 and standard deviation of 0.01 to initialize learning parameters. Adam [22] optimizer is used as the optimization algorithm. We adopt early stopping with the patience of 5 epoch to prevent overfitting, and NDCG@5 is set as the indicator. In particular, we directly use the results of BPR, GMF, GRU4Rec, NARM, CFKG and SLRC reported in Chorus [2]. As for Caser, HGN, SASRec, Locker and KDA, model-specific hyper-parameters for them are set based on the original paper or empirical hyper-parameter search. Specifically, we tune the learning rate in {10^−3^, 10^−4^, 10^−5^}, the *L*_2_ coefficient in {10^−7^, 10^−6^, 10^−5^, 10^−4^}, the dropout in {0.0, 0.1, ⋯, 0.9}. For TRR, the number of historical items *L* used in target-relation regulated representation learning is from {1, 3, 5, 7, 9, 11, 13, 15} and the number of relational items *n* used in constructing query vector is from {1, 2, 3, 4, 5}. We implement our model in PyTorch.

### Experimental results

Table 2 shows the performance comparison of the proposed TRR and other baseline methods on three datasets. We find several observations from this table:

- Sequential models like GRU4Rec and NARM behave better than the traditional collaborative filtering methods (BPR and GMF). This is due to the consideration of sequential dependency. Models like Caser, HGN, SASRec and Locker have achieved extraordinary results due to the high-quality context-aware representations. But they contain more parameters, which requires a lot of data feeding to get a well-trained model. This can be a problem for the sparse user behavior in many datasets.
- The performance of relation-based models like SLRC, Chorus and KDA are among the best baselines. This shows the benefits of exploiting item relations. With regard to KDA, the impressive performance shows the effectiveness of considering item relations and their temporal dynamics by using fourier transform with learnable frequency domain embeddings.
- Finally, we can see that the proposed TRR consistently outperforms the baselines, which confirms the significance of characterizing patterns of item co-occurrence guided by relational items, which transforms the strong correlation imposed by item relations to regulated matching scores between the target and historical items. We conduct t-tests and *p*-value <0.05 proves that the performance improvements of TRR are statistically significant.

**Table 2:** The performance comparison of all methods in three datasets. The best performing method of each column is boldfaced. The second best performing method is underlined.

Methods	Grocery and Gourmet Food				Cellphones and Accessories				Home and Kitchen			
	HR@5	N@5	HR@10	N@10	HR@5	N@5	HR@10	N@10	HR@5	N@5	HR@10	N@10
BPR	0.3242	0.2223	0.4315	0.2571	0.3260	0.2349	0.4364	0.2705	0.2542	0.1718	0.3701	0.2091
GMF	0.3051	0.2089	0.4100	0.2429	0.2866	0.2030	0.3910	0.2367	0.2500	0.1671	0.3693	0.2055
Tensor	0.3478	0.2623	0.4471	0.2943	0.3560	0.2489	0.4888	0.2917	0.2897	0.1941	0.4216	0.2366
GRU4Rec	0.3704	0.2643	0.4721	0.2972	0.4112	0.2956	0.5453	0.3389	0.2953	0.2025	0.4187	0.2423
NARM	0.3590	0.2573	0.4634	0.2910	0.4092	0.2938	0.5440	0.3373	0.2901	0.1976	0.4137	0.2375
Caser	0.3780	0.2690	0.4772	0.3011	0.4194	0.3080	0.5415	0.3474	0.2976	0.2052	0.4129	0.2424
HGN	0.3988	0.2741	0.5233	0.3146	0.4127	0.2993	0.5426	0.3413	0.2952	0.2015	0.4193	0.2416
SASRec	0.4248	0.3043	0.5298	0.3384	0.4622	0.3443	0.5871	0.3848	0.3106	0.2152	0.4329	0.2546
Locker	0.4321	0.3127	0.5357	0.3463	0.4738	0.3539	0.5968	0.3937	0.3160	0.2197	0.4373	0.2588
CFKG	0.4337	0.3081	0.5628	0.3499	0.4465	0.3264	0.5677	0.3656	0.2609	0.1760	0.3801	0.2144
SLRC	0.4513	0.3329	0.5649	0.3698	0.4440	0.3433	0.5414	0.3747	0.3275	0.2452	0.4346	0.2797
Chorus_*BPR*_	0.4754	0.3448	0.5998	0.3852	0.4593	0.3439	0.5784	0.3824	0.3405	0.2473	0.4572	0.2849
Chorus_*GMF*_	0.4748	0.3467	0.5960	0.3861	0.4623	0.3481	0.5809	0.3863	0.3350	0.2461	0.4433	0.2811
KDA	0.5023	0.3727	0.6125	0.4124	0.5288	0.3968	0.6482	0.4403	0.3568	0.2569	0.4759	0.2952
TRR	**0.5257**	**0.3937**	**0.6430**	**0.4317**	**0.5607**	**0.4265**	**0.6788**	**0.4648**	**0.3877**	**0.2868**	**0.5005**	**0.3233**
*p*-value	8.07e-4	6.22e-3	1.31e-4	3.62e-3	1.07e-4	2.22e-3	9.71e-5	1.15e-3	7.39e-3	4.24e-2	2.62e-4	2.39e-2

### Ablation analysis

In this section, we perform a series of ablation experiments on the proposed TRR to confirm its effectiveness and better understand the impact of each key module.

#### Impact of the target-relation regulated representation mechanism

**Fig 3:**
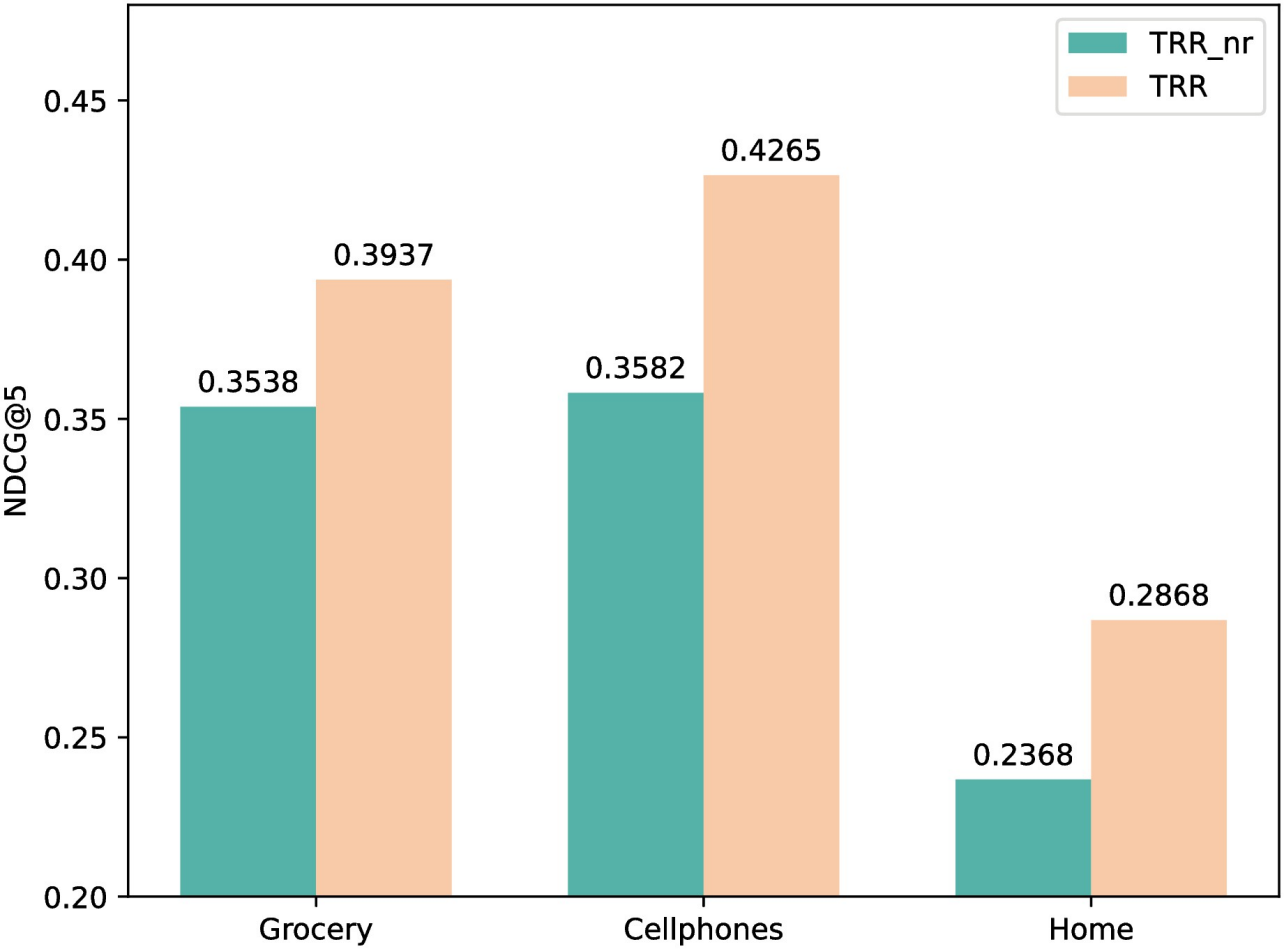
The impact of the target-relation regulated representation learning (NDCG@5).

In order to prove effectiveness of integrating the target and relational items as a whole to characterize the matching scores between the target and historical items, we remove the part of relational items in Eq 4, i.e. only the term ***i*** will be reserved and denote this variant as *TRR*_*nr*. The results are reported in Fig 3. The performance drop of *TRR*_*nr* demonstrates the advantage of mining the strong correlation between the target and relational items. It exploits the co-occurrence of three items, thus leading to more accurate scoring.

#### Impact of gating layer

**Fig 4:**
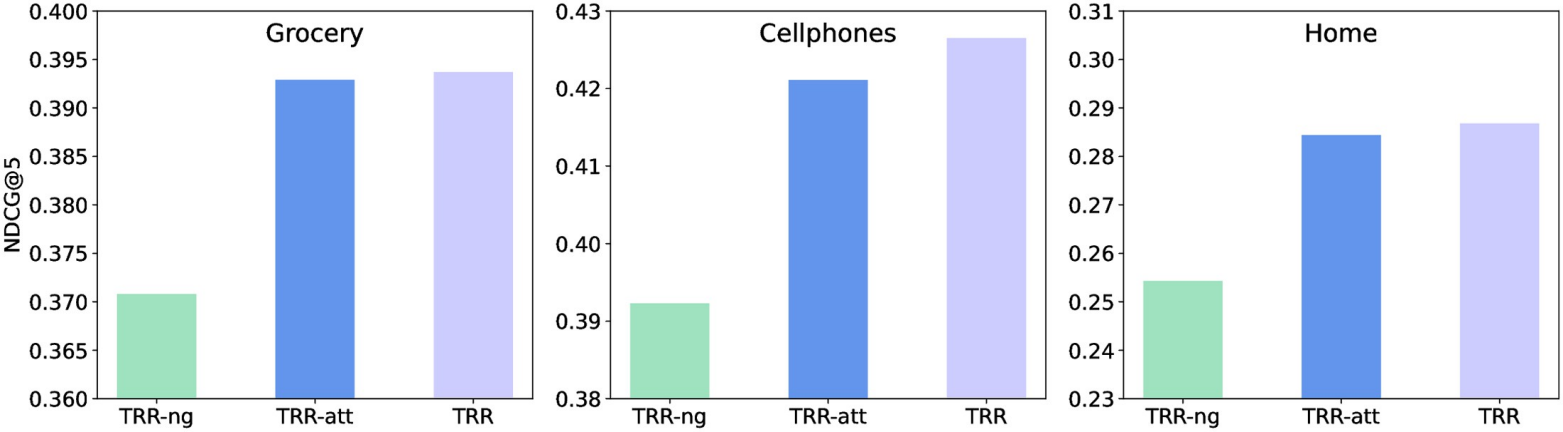
The impact of the gating layer on three datasets (NDCG@5).

Our proposed approach TRR leverages a gating layer in the target-relation regulated mechanism to adjust the weight of matching degrees between the target and historical items, which is done in Eq 9. We study the impact of this gating layer by comparing two variants. The first variant is by removing the gating layer from TRR and denoted as TRR-ng. The second variant is by replacing the sigmoid function in Eq 9 with softmax and denoted as TRR-att. The results are reported in Fig 4. The performance degradation in TRR-ng indicates that it is helpful to further adjust the relative scales within the group of historical items through the gating mechanism. This can improve the distinguishability of important items. Besides, we can see that the overall performance of TRR-att is slightly inferior compared to TRR. The gap is more obvious on the Cellphones dataset. This is because in this dataset, there are more items with important relations in user’s behavior sequence, which can be seen from the column “relational ratio in test set” in Table 1. Sigmoid is better compatible with this scenario.

#### Impact of training method for knowledge graph

We now evaluate the influence of training methods on knowledge graph. Three additional knowledge graph embedding models are compared here with the adopted TransE [19] in TRR, which are TranH [23], ConvR [24], and RotateE [25]. TransH is a translational model where entities have different representations under different relations. ConvR utilizes a convolutional network designed to maximize entity-relation interactions. RotatE defines each relation as a rotation from the source entity to the target entity in the complex vector space. We denote the three models using them as “TRR w TransH”, “TRR w ConvR” and “TRR w RotatE” respectively in Table 3. From the results, we can note that newer models like RotatE and ConvR are inferior to the old ones like TransH and TransE (adopted in our model TRR). This can be attributed to the simple relationships in the constructed knowledge graph, where the number of edge types is small. In such case, complex models are prone to overfitting and thus we prefer simple models with fewer parameters.

**Table 3:** The impact of four different training methods for knowledge graph embedding (NDCG@5).

Architecture	*Grocery and Gourmet Food*	*Cellphones and Accessories*	*Home and Kitchen*
TRR w TransH	0.3902	0.4248	0.2869
TRR w ConvR	0.3634	0.3764	0.2616
TRR w RotatE	0.3925	0.4150	0.2489
TRR	0.3937	0.4265	0.2868

#### Impact of relational item number *n*

**Fig 5:**
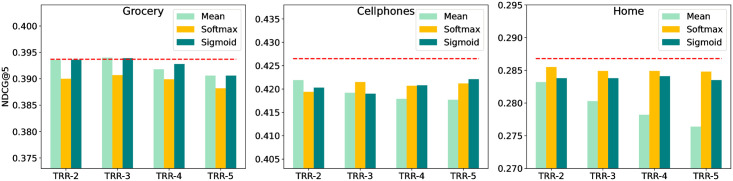
The impact of different relational item number *n* within three aggregating methods (NDCG@5).

To study the effect of different number of relational items incorporated in Eq 4, we vary *n* in the range of 1 to 5 and use the three aggregation methods described there. The results using Eq 5 are denoted as Mean, the results using Eq 6 as Softmax and the results using Eq 7 as Sigmoid. The performance is reported in Fig 5, where TRR-2 sets *n* to 2, TRR-3 sets *n* to 3 and so on. The proposed TRR adopts *n* = 1 and λ_1_ = 1 and the results are marked with red dotted line. We can observe that different datasets have their own suitable aggregation methods, which are determined by the distribution characteristics of datasets. In general, more relational items will cause performance degradation in many cases. This is reasonable since the correlation between relational items often weakens over time. The older the relational item is, the less meaningful it is for the target item, and the possibility of introducing noise is increased instead.

### Influence of hyper-parameter

**Fig 6:**
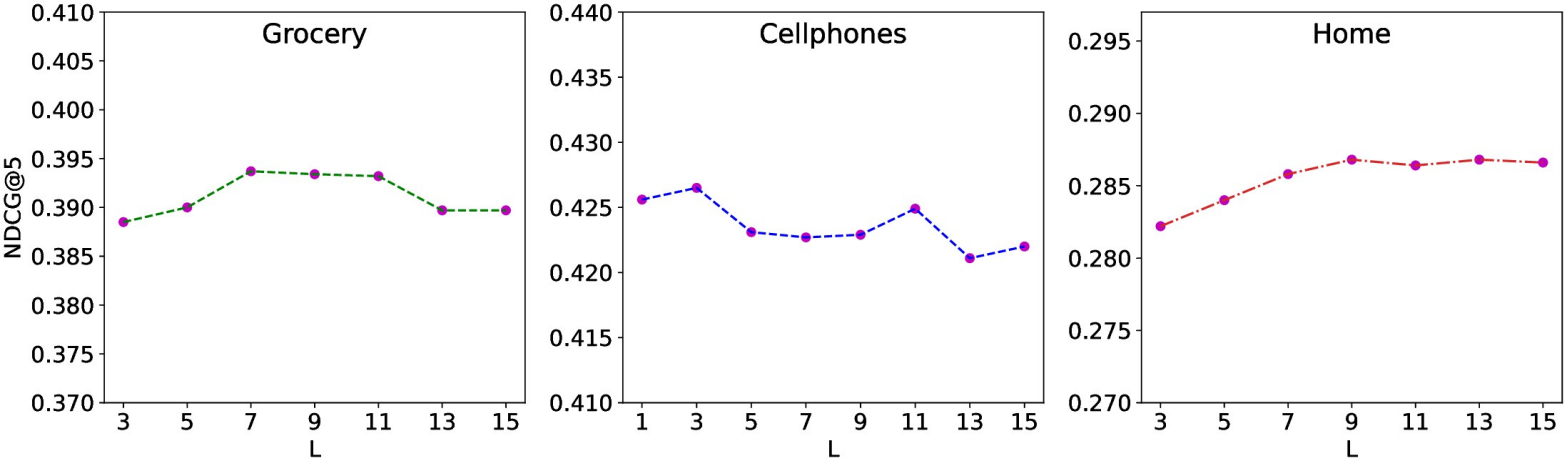
The impact of different history length *L* (NDCG@5).

Proper setting of hyper-parameter is important for model performance. In this section, we evaluate the proposed TRR to investigate the impact of history length *L* in our model, which is used to calculate the matching scores between the target and historical items in the target-relation regulated representation learning mechanism. The results are shown in Fig 6 measured with NDCG@5. According to the results, we can observe that it’s not good if *L* is too large or too small. Small *L* leads to insufficient context information while large value will cause interference from many irrelevant items. The best setting varies for different datasets, which depends on the data size and sparsity. In addition, the results do not fluctuate much near the appropriate length, which shows the stability and robustness of our model in delivering superior prediction accuracy.

## Conclusion

In this work, we focus on employing the correlation between the target and relational items and propose a target-relation regulated mechanism TRR for sequential recommendation with item relations. The strong relationships between them extend to the calculation of matching degrees and are informative to the pattern of item co-occurrence. TRR utilizes such knowledge and integrates relational items to the calculation of matching scores between the target and historical items. Extensive experimental results and analysis on three real-world datasets demonstrate that our proposed model TRR consistently outperforms the state-of-the-art methods.

Due to the data sparsity, Relations may actually exist between some items but not observed. The performance improvement of our TRR is dependent on these relational items however. In the future, we plan to extend TRR to incorporate other auxiliary information to enrich relational items of the target item. Auxiliary information such as category, user review and knowledge base can be employed to get more appropriate relational items from different perspectives.

## Supporting information

S1 File (ZIP)

## References

[1] Zhang Y, Ai Q, Chen X, Wang P. Learning over knowledge-base embeddings for recommendation. arXiv preprint arXiv:180306540. 2018.

[2] Wang C, Zhang M, Ma W, Liu Y, Ma S. Make It a Chorus: Knowledge-and Time-aware Item Modeling for Sequential Recommendation. In: Proceedings of the 43rd International ACM SIGIR; 2020. p. 109–118.

[3] Loyola P, Liu C, Hirate Y. Modeling user session and intent with an attention-based encoder-decoder architecture. In: Proceedings of the Eleventh ACM Conference on Recommender Systems; 2017. p. 147–151.

[4] Rendle S, Freudenthaler C, Gantner Z, Schmidt-Thieme L. BPR: Bayesian personalized ranking from implicit feedback. arXiv preprint arXiv:12052618. 2012.

[5] Koren Y. Factorization meets the neighborhood: a multifaceted collaborative filtering model. In: Proceedings of the 14th ACM SIGKDD international conference on Knowledge discovery and data mining; 2008. p. 426–434.

[6] He X, Liao L, Zhang H, Nie L, Hu X, Chua TS. Neural collaborative filtering. In: Proceedings of the 26th international conference on world wide web; 2017. p. 173–182.

[7] Hidasi B, Karatzoglou A, Baltrunas L, Tikk D. Session-based recommendations with recurrent neural networks. arXiv preprint arXiv:151106939. 2015.

[8] Tang J, Wang K. Personalized top-n sequential recommendation via convolutional sequence embedding. In: Proceedings of the Eleventh ACM International Conference on Web Search and Data Mining; 2018. p. 565–573.

[9] Pei W, Yang J, Sun Z, Zhang J, Bozzon A, Tax DM. Interacting attention-gated recurrent networks for recommendation. In: Proceedings of the 2017 ACM on Conference on Information and Knowledge Management; 2017. p. 1459–1468.

[10] Mei L, Ren P, Chen Z, Nie L, Ma J, Nie JY. An attentive interaction network for context-aware recommendations. In: Proceedings of the 27th ACM International Conference on Information and Knowledge Management; 2018. p. 157–166.

[11] Ying H, Zhuang F, Zhang F, Liu Y, Xu G, Xie X, et al. Sequential recommender system based on hierarchical attention network. In: IJCAI International Joint Conference on Artificial Intelligence; 2018.

[12] He Z, Zhao H, Lin Z, Wang Z, Kale A, Mcauley J. Locker: Locally Constrained Self-Attentive Sequential Recommendation. In: Proceedings of the 30th ACM International Conference on Information & Knowledge Management; 2021. p. 3088–3092.

[13] Ma C, Kang P, Liu X. Hierarchical gating networks for sequential recommendation. In: Proceedings of the 25th ACM SIGKDD; 2019. p. 825–833.

[14] Kang WC, McAuley J. Self-attentive sequential recommendation. In: 2018 IEEE International Conference on Data Mining (ICDM). IEEE; 2018. p. 197–206.

[15] Kang WC, Wan M, McAuley J. Recommendation through mixtures of heterogeneous item relationships. In: Proceedings of the 27th ACM International Conference on Information and Knowledge Management; 2018. p. 1143–1152.

[16] Park C, Kim D, Oh J, Yu H. Do “Also-Viewed” Products Help User Rating Prediction? In: Proceedings of the 26th International Conference on World Wide Web; 2017. p. 1113–1122.

[17] Wan M, Wang D, Liu J, Bennett P, McAuley J. Representing and recommending shopping baskets with complementarity, compatibility and loyalty. In: Proceedings of the 27th ACM International Conference on Information and Knowledge Management; 2018. p. 1133–1142.

[18] Wang C, Ma W, Zhang M, Chen C, Liu Y, Ma S. Toward Dynamic User Intention: Temporal Evolutionary Effects of Item Relations in Sequential Recommendation. ACM Transactions on Information Systems (TOIS). 2020;39(2):1–33.

[19] Bordes A, Usunier N, Garcia-Duran A, Weston J, Yakhnenko O. Translating embeddings for modeling multi-relational data. Advances in neural information processing systems. 2013;26:2787–2795.

[20] Karatzoglou A, Amatriain X, Baltrunas L, Oliver N. Multiverse recommendation: n-dimensional tensor factorization for context-aware collaborative filtering. In: Proceedings of the fourth ACM conference on Recommender systems; 2010. p. 79–86.

[21] Wang C, Zhang M, Ma W, Liu Y, Ma S. Modeling item-specific temporal dynamics of repeat consumption for recommender systems. In: The World Wide Web Conference; 2019. p. 1977–1987.

[22] Kingma DP, Ba J. Adam: A Method for Stochastic Optimization. In: Proceedings of the 4th International Conference on Learning Representations (ICLR); 2015. p. 1–15.

[23] Wang Z, Zhang J, Feng J, Chen Z. Knowledge graph embedding by translating on hyperplanes. In: AAAI. vol. 14. Citeseer; 2014. p. 1112–1119.

[24] Jiang X, Wang Q, Wang B. Adaptive convolution for multi-relational learning. In: Proceedings of the 2019 Conference of the North American Chapter of the Association for Computational Linguistics; 2019. p. 978–987.

[25] Sun Z, Deng ZH, Nie JY, Tang J. Rotate: Knowledge graph embedding by relational rotation in complex space. arXiv preprint arXiv:190210197. 2019.

